# Fermented Grapevine Leaves: Potential Preserving Agent in Yogurt

**DOI:** 10.3390/foods13132053

**Published:** 2024-06-27

**Authors:** Lais Freitas, Miguel Sousa-Dias, Vanessa B. Paula, Luís G. Dias, Leticia M. Estevinho

**Affiliations:** 1 Doctoral School, University of León (ULE), Campus de Vegazana, 24007 León, Spain; laisfreitas@ipb.pt (L.F.); vanessapaula@ipb.pt (V.B.P.); 2 Centro de Investigação de Montanha (CIMO), Instituto Politécnico de Bragança, Campus de Santa Apolónia, 5300-252 Bragança, Portugal; miglsdias@gmail.com (M.S.-D.); ldias@ipb.pt (L.G.D.); 3 Laboratório para a Sustentabilidade e Tecnologia em Regiões de Montanha, Instituto Politécnico de Bragança, Campus de Santa Apolónia, 5300-253 Bragança, Portugal

**Keywords:** *Vitis vinifera*, fermentation, yogurt, preserving, *Saccharomyces cerevisiae*

## Abstract

In this study, we monitored the fermentative process of *Vitis vinifera* L. leaves (grapevine), spontaneously or promoted by *Saccharomyces cerevisiae*, in both solid and liquid media. We also aimed to evaluate the effect on the bioactivity and shelf life of yogurt incorporating fermented and non-fermented grapevine leaves compared to yogurt produced with the preservative potassium sorbate. The results revealed that fermented grapevine leaf extracts increased their bioactive compounds and antioxidant activity, particularly in fermentations in a solid medium. In yogurt samples with incorporation extract from solid spontaneous fermentation and extract from solid yeast fermentation, even in small quantities, they exhibited higher levels of total phenols (1.94 and 2.16 mg GAE/g of yogurt, respectively) and antioxidant activity (5.30 and 5.77 mg TroloxE/g of yogurt; and 1.33 and 1.34 mg Fe(II)E/g of yogurt, respectively) compared to control yogurt (1.44 mg GAE/g of yogurt, 4.00 mg TroloxE/g of yogurt, and 1.01 mg Fe(II)E/g of yogurt). Additionally, yogurts supplemented with fermented grapevine leaves demonstrated the potential to inhibit microbial growth without impairing the multiplication of lactic acid bacteria.

## 1. Introduction

Yogurt is a fermented milk product that has become popular over the years for its nutritional profile, stability, natural characteristics and status as a healthy food. Yogurt, as well as other fermented dairy products, is obtained through fermentation by lactic acid bacteria, which are mainly responsible for the product’s characteristics, such as its taste, aroma, and texture. This technology consists of inoculating pasteurized milk with lactic acid bacteria, *Streptococcus thermophilus* and *Lactobacillus bulgaricus*. Fermentation begins with the production of lactic acid by *S. thermophilus* and then, *L. bulgaricus* is used to provide the characteristic flavor of yogurt. Studies show that the use of these bacteria has health benefits due to the fact that they stimulate the immune system and regulate the gastrointestinal system [1,2].

During the fermentation process, due to the enzymatic action of lactic acid bacteria, biochemical reactions take place that are responsible for converting organic substances into smaller compounds [3].

Yogurt is a product in which functional additives or those with greater nutritional value can be incorporated to enrich the food and, consequently, have beneficial effects on consumer health. In the case of extending the shelf life of food, additives are used that are able to preserve the food by inhibiting the multiplication of microflora, thus preventing it from deteriorating [4,5].

Sorbic acid is an additive widely used as a preservative in the food industry, as it is mainly capable of inhibiting the growth of molds and yeasts [6]. However, the use of these additives should be moderated as they can pose a risk to consumer health [4]. The maximum limit of potassium sorbate allowed by Regulation (EU) n° 1129/2011 on food additives in yogurt is 1000 mg/kg [7].

In recent years, the demand for healthier foods with fewer chemical additives and preservatives and with functional properties capable of promoting health benefits has increased considerably, as is the case with probiotic foods and natural additives [3,8]. In this context, a recently studied alternative is the use of vine leaves, which are an abundant source of vitamins and phenolic compounds [8].

*V. vinifera* L., native to Western Asia and Europe, produces fruit that is widely used for both culinary and beverage purposes [9]. Beyond the primary yield of grapes, *V. vinifera* L. offers an array of ancillary products and by-products, such as grape leaves and pomace [10]. Grape leaves, in particular, are rich in phenolic compounds that are known for their powerful antioxidant and anti-inflammatory properties. These bioactive constituents, including polyphenols, resveratrol, quercetin, anthocyanins, flavanols, vitamins, and minerals, offer significant health benefits and can significantly enhance the functional properties of foods [11]. The biological properties of grapevine leaves have been widely recognized, mainly due to their rich phenolic compounds, including phenolic acids, flavonols, tannins, and anthocyanins. These compounds, predominantly found in the leaves, provide liver protection and exhibit a range of beneficial effects, such as their antibacterial, antifungal, antiviral, anti-inflammatory, and antioxidant activities. The use of grape leaves, a by-product of viticulture, promotes sustainability and adds value to agricultural waste, supporting the principles of a circular economy. The extraction of bioactive compounds from *Vitis* leaves represents a promising solution for the management of ecotoxic waste. It also facilitates the development of innovative and valuable natural products with multiple applications in different industries by exploiting their bioactive properties [9]. A major advantage of yogurt is that it can be used as a natural additive to food products. Yogurt, a widely consumed and globally accepted dairy product, offers an ideal medium for introducing new functional ingredients without changing consumers’ eating habits. Its matrix is particularly suitable for incorporating bioactive compounds, ensuring controlled release, and improving the bioavailability of these phenolic compounds [12].

Fermentation by yeasts is a processing technique widely used in the food industry as it is responsible for transforming starch and sugars into alcohol and CO_2_, resulting in products such as beer, wine, and bread. This process has the advantage of low energy costs and allows enzymes such as lipase, amylase, glucoamylase, and protease to synthesize bioactive compounds present in the substrate [13].

Yeasts are also used for their functional capacity as stabilizers and thickeners. The most commercially used genera are *Saccharomyces* and *Candida*. Each strain requires specific medium, temperature, and aeration conditions for growth [14,15]. Fermentation can cause biological changes in the substrates, producing biologically active secondary metabolites and increasing their antioxidant activity. In previous studies, researchers have observed that fermentation in solid media can increase the antioxidant content and nutritional profile of cereals and legumes [16,17].

The study carried out by Dhull et al. [16] indicated that fermentation conducted by *Aspergillus awamori* in a solid state is an efficient, economical, and reliable method, as it promotes an increase in total phenolic content and condensed tannin content. García and Bianchi [18] indicated that solid-medium fermentation with the fungus *Penicillium purpurogenum* was able to increase the content of phenolic compounds in extracts obtained from coffee husks. According to Kosar et al. [19], the content of total flavonoids, such as quercetin, can be increased by fermenting the leaves.

In view of the above, no studies have been found in the literature regarding the introduction of fermented vine leaf extract for enrichment or as a natural additive in foods.

This work focused on two main objectives. First, it focused on evaluating the ability of vine leaf extracts fermented in solid or liquid media to fully or partially replace potassium sorbate, the conventional preservative of yogurt. Secondly, we investigated whether yogurt enriched with fermented leaf extracts could provide additional health benefits due to their high levels of bioactive compounds. Given the high concentration of bio-active compounds in leaves, it was also investigated whether incorporating leaf extracts into yogurt could enhance its health benefits.

## 2. Materials and Methods

### 2.1. Sampling

The leaves of *V. vinifera* L. were harvested at the Agrarian School (ESA) of the Polytechnic Institute of Bragança, Portugal, in June 2020. The leaves were harvested during the vegetative cycle of the vine, when the plant has little water availability. Under these conditions, the plant becomes water stressed and concentrates on the production of high-quality and concentrated by-products, such as the 3-O-glycosides of campherol and quercetin [9].

Undamaged leaves were selected, weighed, and dried in a forced-air oven (VWR^®^ VENTI-Line^®^, Carnaxide, Portugal; ±0.1 °C) at 40 °C for approximately 72 h to a constant weight. The dried leaves were ground in an IKA TUBE-MILL (Deutschland, Germany) mill until a powder was obtained (grain size 250–800 mesh).

### 2.2. Reagents

The *Saccharomyces cerevisiae* culture belonged to the microorganism collection of the Escola Superior de Agrária de Bragança, Portugal (ESA).

Yeast extract was obtained from HiMedia Laboratories (Modautal, Germany), bacteriological peptone and glucose were obtained from OXOID LTD (Basingstoke, Hampshire, UK), and glucose (dextrose) monohydrate was obtained from CeaMed, Lda CEIM (Funchal, Portugal). Rose Bengal CAF agar culture medium was supplied by Liofilchem (Roseto degli Abruzzi, Italy), and Man, Rogosa and Sharpe (MRS) agar culture medium was supplied by HiMedia (Mumbai, India). Modified iron sulfite agar culture medium was supplied by HiMedia Laboratories (Modautal, Germany). The SimPlate for Total Plate Count (TPC) kit was provided by Biocontrol^®^ (Bellevue, WA, USA). Baird-Parker medium (BP) was provided by HiMedia Laboratories (Modautal, Germany). Nutrients were provided by Enovit^®^ of the AEB Group (Viseu, Portugal).

Ethanol, 99.8% pure, was obtained from Carlo Erba Reagents (Chaussee du Vexin, France). Folin–Ciocalteau, DPPH, aluminum chloride, sodium carbonate, quercetin, gallic acid, Trolox, FRAP, acetic acid, TPTZ, FeCl_3_, FeSO_4_.7H_2_O, potassium sorbate, ammonium nitrate (NH_4_NO_3_), sulfur dioxide, and the other reagents were purchased from Sigma Chemical Co. (St. Louis, MO, USA). Sulfuric acid (H₂SO₄), sodium hydroxide (NaOH), and copper sulfate (CuSO₄) were obtained from Merck KGaA (Darmstadt, Germany); mercury sulfate (HgSO₄), hydrochloric acid (HCl), and potassium sulfate (K₂SO₄) were obtained from Scharlau (Barcelona, España). Sodium chloride (NaCl) was provided by Pronalab (Lisbon, Portugal). Light petroleum was supplied by Chem-LAB (Zedelgem, Belgium).

### 2.3. Fermentations

Liquid medium fermentation: A liquid spontaneous control fermentation (LSF) of the leaves was conducted alongside another liquid fermentation inoculated with *Saccharomyces cerevisiae* yeast (LYF), following the methodology reported by Ferreira et al. [20]. In the initial experiment, 10% and 30% glucose/g of leaves were utilized. Based on the results obtained from the first fermentation, the subsequent experiment proceeded with 15% and 20% glucose/g of sample to optimize the conditions of the fermentation process. The liquid medium fermentation involved preparing a medium of 90.00 mL of saline solution (0.85%), 0.60 g/L of commercial nutrients (Enovit—AEB Group), and 10.00 mL of distilled water. To achieve a sterile medium, autoclaving was performed (121 °C for 60 min). After cooling, 3 g of the leaf sample was introduced, followed by immersion in a water bath (80 °C for 5 min) and a thermal shock in ice. Finally, 125 μL of 6% sulfur dioxide per 100 g of sample and *Saccharomyces cerevisiae* yeast (1 × 10^6^ CFU/g) was added from the collection of microorganisms belonging to ESA. In the case of LSF, neither pasteurization nor inoculum addition was carried out. The experiment was conducted for 14 days at 25 °C. To monitor the fermentations, the following parameters were evaluated every 24 h: flask weight, Brix degree, and assessment of colony number/mL on YPD solid medium. In the latter case, the results were expressed in natural logarithm colony-forming units per milliliter of sample (ln CFU/mL).

Solid medium fermentation: To assess the behavior of *Saccharomyces cerevisiae* in solid-state medium, two fermentations were conducted, one spontaneous (SSF) and one inoculated (SYF), using the methodology described by Dulf et al. [21] with slight adaptations. The medium was prepared using a 0.003% saline solution, 2.0 g of glucose, and 2.0 g of NH_4_NO_3_. The solution was autoclaved (121 °C for 60 min), and after cooling, 30.0 g of leaf sample was introduced. It underwent pasteurization and thermal shock, and finally, the inoculum was introduced (1 × 10^6^ CFU/g of leaves). Fermentation took place for 9 days at 25 °C. Microbiological control was also assessed by plating on YPD medium at days 1, 3, and 9. From the plate counts on YPD solid medium, the number of natural logarithm colony-forming units per gram of sample (ln CFU/g) was determined. A linear regression analysis was performed using Microsoft Corporation’s Excel program (2016) with the natural logarithm values of CFU/g (ln CFU/g) versus time, which are not presented in this work. From the exponential growth phase, the log phase, the equation of the line was obtained to determine the specific growth rate (µ_c_) for each of the fermentations. The doubling time (DT) was calculated by dividing ln2 by the specific growth rate (µ_c_).

### 2.4. Extract Procedure

To prepare the extract from liquid medium fermentation, the fermented products were first placed in sterile tubes and centrifuged (5810 R, New York, NY, USA) to separate the solid part. The supernatant was then evaporated to remove part of the alcohol obtained in the fermentation process and subsequently lyophilized (Labconco, Freezone 4.5 model, Kansas City, MO, USA). The extracts were obtained by double hydroalcoholic extraction with 80% ethanol, as described by Paula [22] followed by drying in a rotary evaporator (IKA RV8, Deutschland, Germany) at 40 °C. For sample preparation, 0.1 g of extract (4 mg/mL) was added to a 25 mL volumetric flask with 80% ethanol.

### 2.5. Yogurt Production

For yogurt production, natural yogurt (Continente) was used as an inoculum at a ratio of 120 g/L of milk (12.0%, *w*/*v*). The natural yogurt used as the inoculum contained a concentration of lactic acid starter cultures (live bacterial cultures) of approximately 10^8^ CFU/g. These starter cultures consist of *L. bulgaricus* and *S. thermophilus* bacteria. They are essential for fermenting milk and converting lactose into lactic acid, which gives yogurt its characteristic texture and flavor. In fact, Celik and Temiz [23] report that wild strains are able to produce quantitatively and/or qualitatively different flavor compounds that give yogurt its own desirable characteristics. Initially, 1 L of ultra-high temperature processing (UHT; Continente) whole milk (3.6% fat) was boiled along with sugar (12.6%, *w*/*v*) and powdered milk (1.0% fat; 1.0%, *w*/*v*). We used UHT milk because this type of milk is the most available in our country and has been used in several prior studies focused on yogurt production [24].

To inoculate the yogurt, the sample needed to be cooled to 42 ± 2 °C. The sample was then conditioned in a hermetic container in a thermostat bath for 8 h at 42 ± 2 °C (to reach the pH 4.6, which is required for the product to curdle) [25], followed by placement in a refrigerator (4 ± 2 °C) for 48 h. After this period, the coagulum was broken with a sterile glass rod, and pasteurized strawberry pulp (10.0%, *w*/*v*) was added. The process of obtaining strawberry pulp involved cooking only sanitized and frozen strawberries over medium heat until they reached a gelatinous texture, followed by storage in a sterile container. The extracts that were added to the yogurt were prepared as described in Section 2.4. The yogurts were filled in a bio safety cabinet (Microflow Class 2, Gravesend, UK) packaged in sterile Schott bottles with lids as follows: control yogurt (Cy) with potassium sorbate (0.1%, *w*/*v*); yogurt SSFy with extract from spontaneous solid-state fermentation (0.1%, *w*/*v*); yogurt SYFy with extract from yeast solid-state fermentation (0.1%, *w*/*v*); and yogurt LYFy with extract from liquid-state yeast fermentation (0.1%, *w*/*v*). A different yogurt was used for each analysis timepoint: one (1 day), second (7 days), and third (12 days). Each sample preparation was performed with 80 g in triplicate and refrigerated (4 ± 2 °C) for subsequent analysis.

### 2.6. Physicochemical Properties

The physicochemical composition, including the ash and lipid content of grapevine leaves and fermentations, was evaluated. The ash content was determined in triplicate by incinerating samples in a muffle furnace at 550 °C (Lindberg, model 51894, Atlanta, GA, USA; ±1.5 °C). The quantification of mineral residue was calculated by the difference between the mass sample and the mass of the ash. Total lipid content was determined using the continuous extraction method using a Soxhlet apparatus (Behrotest, Labor-Technik, Düsseldorf, Germany). All of the analyses were conducted in accordance with AOAC [26] guidelines.

For yogurt samples, analyses of proteins, ash, lipids, pH, and titratable acidity were performed at days 1, 7, and 12. Ash and lipid analyses were performed as described above. The pH of the yogurt samples was measured using a Potentiometric (Mettler Toledo Model, Mumbai, India) with a combined pH electrode. The percentage of lactic acid in yogurt was quantified using titration with NaOH (0.08 N), with phenolphthalein as the indicator. The Kjeldahl method was used for protein quantification and total nitrogen content [26].

### 2.7. Bioactive Compounds and Antioxidant Activity

The analysis of bioactive compounds and antioxidant activity was carried out both for the leaves and their fermented products and for the yogurts enriched with the extracts. The leaf extracts and their fermentations were prepared according to Section 2.4. Yogurt samples also underwent the drying and concentration process in a lyophilizer for subsequent extraction, according to Section 2.4.

To determine the total phenolic content, the methodology adapted from Singleton, Orthofer, and Lamuela-Raventos [27] was applied, using gallic acid equivalents (GAEs) as the standard. The results were expressed as the mg of GAE/g of a sample, as obtained from the calibration curve (y = 0.0098x + 0.0081; R^2^ = 0.999).

The flavonoid content was determined according to the methodology reported by Savi et al. [28], using quercetin (QE) as the standard. Results were expressed as mg of QE/g of sample, as obtained from the calibration curve (y = 0.027x − 0.082; R^2^ = 0.996).

The ferric-reducing ability (FRAP) was quantified following the methodology employed by Santos et al. [29], where ferrous sulfate served as the standard. The results were expressed in mg Fe(II)E/g of sample, as obtained from the calibration curve (y = 0.0026x − 0.05591; R^2^ = 0.999).

The DPPH assay was performed based on the work of Paula [22], using Trolox as the standard. Results were expressed as mg TroloxE/g of sample, as obtained from the calibration curve (y = 1.307x − 2.829; R^2^ = 0.997). The concentration of DPPH was calculated based on its blocking effect using the following equation:

% inhibition = [(Abs _DPPH_ − Abs _S_)/Abs _DPPH_] × 100 (1)

where % inhibition = percentage of antioxidant activity; Abs _DPPH_ = solution absorbance with the sample and the free radical DPPH; Abs _S_ = absorbance of the solution with sample without the free radical DPPH.

### 2.8. Microbiological Analysis

To determine microbiological quality, the resulting products from fermentations were evaluated on days 1, 3, and 9, and yogurt samples were assessed at days 1, 7, and 12. Sample preparation involved adding 1 g of sample with buffered water (0.1%) to a sterile tube, resulting in a concentration of 10^−1^ g/mL. Successive dilutions were then made as necessary to obtain the result for each analysis.

The quantification of molds and yeasts was performed using Rose Bengal CAF Agar medium, Liofilchem, Italy [30], following the manufacturer’s instructions. Growth was observed from the second day until day 7 of incubation at 25 °C.

The methodology for lactobacilli counting involved immersing the sample in a double layer of MRS Agar (Man, Rogosa, and Sharpe), as described by Ferreira et al. [20]. Plates were covered with Parafilm and incubated at 30 °C for 72 h.

The analysis for the detection of sulfite-reducing clostridia spores was based on the AOAC manual [26]. Using aseptic conditions, the samples were immersed in a double layer of Modified Iron Sulphite Agar (HIMEDIA) in test tubes to ensure anaerobiosis. These tubes were covered with Parafilm and incubated at 37 °C for 5 days.

The SimPlate kit (Biocontrol^®^) was used for the determination of coliforms at 37 °C and *Escherichia coli* according to the manufacturer’s specifications. Total coliform presence was quantified by color change from blue to pink, and *E. coli* detection and quantification were conducted by counting wells exhibiting fluorescence under exposure to ultraviolet (UV) light at 365 nm.

Following the methodology used by Santos et al. [29], coagulase-positive staphylococci counting was performed on Baird-Parker medium (BP-HIMEDIA) supplemented with potassium tellurite and egg yolk saline solution. Incubation was carried out for 48 h at 37 °C.

Overall, microorganism counts were expressed as colony-forming units (CFUs) per gram or milliliter of sample. Only the result for sulfite-reducing Clostridium was indicated by presence or absence.

### 2.9. Statistical Analysis

Overall, the assumptions for each ANOVA analysis confirmed that the data showed homogeneity of variances (Levene’s test *p*-value > 0.05) and normality (Shapiro–Wilk test *p*-value > 0.05). To compare the samples, the results underwent one-way ANOVA (analysis of variance) followed by Tukey’s test (5.0%), utilizing SPSS software (version 20.0).

## 3. Results and Discussion

### 3.1. Fermentations

#### 3.1.1. Liquid Media Fermentation

**Figure 1:**
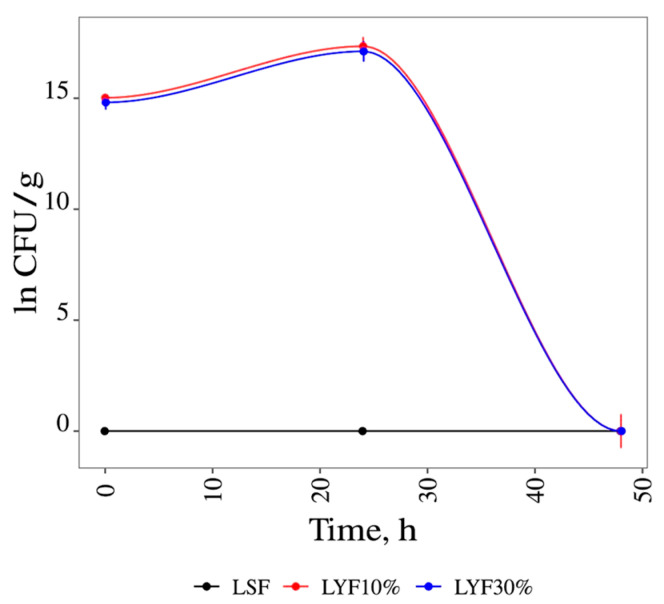
Fermentation with 10% and 30% glucose. LSF = liquid spontaneous fermentation; LYF10% = liquid yeast fermentation with 10% of glucose; LYF30% = liquid yeast fermentation with 30% of glucose.

In this study, it was found that *Saccharomyces cerevisiae* grown in a culture medium containing grapevine leaves (1 × 10^6^ CFU/g of leaves) and 10% glucose was identical to that observed in a medium in which the glucose concentration was increased to 30%, as can be seen in Figure 1.

It can be seen in Figure 1 that, in both cases, fermentation terminus occurred at 48 h. To increase the duration of the fermentation process, other concentrations of glucose were tested.

This study revealed that no microbial growth occurred during the 48 h of spontaneous fermentation. This suggests that the result obtained was likely due to a lack of nutrients (carbon source). In this circumstance, glucose was added in subsequent experiments. Based on these results, it was decided to use 15% and 20% glucose/g of sample in both yeast-inoculated (*S. cerevisiae*) and spontaneous fermentation.

The weight and °Brix (%) of the samples were also monitored to follow the performance of the fermentations (shown in Figures S1 and S2 of the Supplementary Materials, respectively).

It was observed that the weight of the flasks decreased over time as a consequence of substrate (glucose) consumption during fermentation. However, spontaneous fermentation with 20% glucose/g sample (LSF20%) exhibited less weight loss, and the °Brix (%) showed no variation, indicating that even with 20% substrate, spontaneous fermentation of the leaves practically did not occur.

From the analysis of the figure, it is noted that the other fermentations (LSF15%, spontaneous fermentation with 15% of glucose, LYF15%, yeast fermentation with 15% of glucose, and LYF20%, yeast fermentation with 20% of glucose) exhibited a similar behavior regarding the reduction in flask weight and °Brix (%) throughout the fermentation process, suggesting substrate consumption by *S. cerevisiae*.

**Figure 2:**
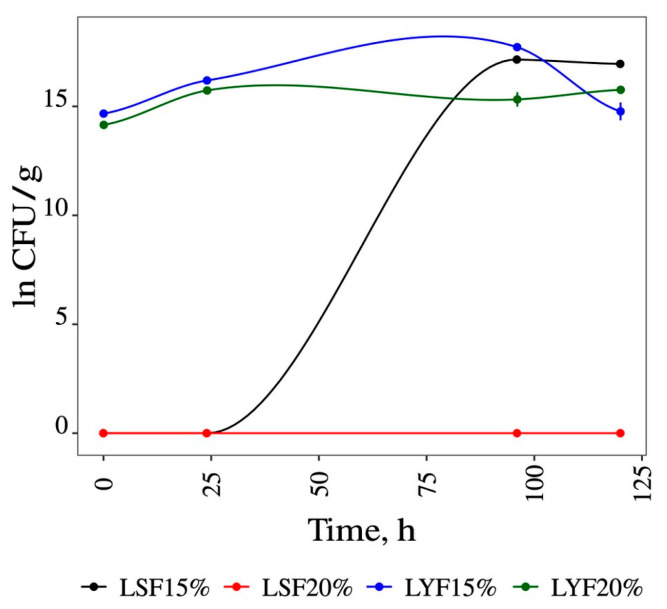
Variation of the natural logarithm of CFUs over time for *Saccharomyces cerevisiae* grown in liquid medium with 15% and 20% glucose. LSF15% = liquid spontaneous fermentation with 15% of glucose; LSF20% = liquid spontaneous fermentation with 20% of glucose; LYF15% = liquid yeast fermentation with 15% of glucose; LYF20% = liquid yeast fermentation with 20% of glucose.

Figure 2 illustrates the variation in the ln of colony-forming units (CFUs) as a function of time in the various experimental conditions over the fermentation time. The results indicate that in fermentations containing 15% glucose (LSF15% and LYF15%), yeast growth was higher than in fermentations containing 20% glucose (LSF20% and LYF20%); therefore, in subsequent studies, only 15% glucose/g sample was used.

**Figure 3:**
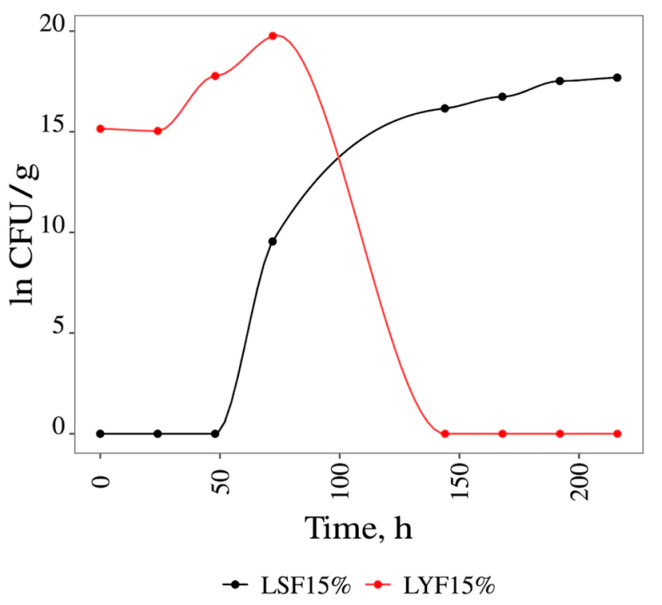
Variation in the ln of the CFU related with time of *S. cerevisiae* grown in liquid media with 15% of glucose. LSF15% = liquid spontaneous fermentation with 15% of glucose; LYF15% = liquid yeast fermentation with 15% of glucose.

The results obtained in the analysis of °Brix (%) and, consequently, the consumption of sugars using 15% glucose in all the fermentations are shown (see Figure S3, Supplementary Materials). These figures demonstrate that both fermentations started 24 h after inoculation, as there was a reduction in °Brix (%) and an increase in final biomass over time. Figure 3 shows that in spontaneous fermentation (LSF), the final biomass obtained at 216 h of fermentation was 17.69 ln CFU/mL. This growth was due to the microbiota present on the leaf. Meanwhile, in *S. cerevisiae* fermentation, the final biomass was 19.76 ln CFU/mL, and fermentation ended 72 h after inoculation. After, the °Brix (%) remained constant.

From the results obtained for the variation in CFU, the speed (rate) of growth of *S. cerevisiae* was calculated under all the experimental conditions. Table 1 shows the values obtained for the Lag phase (h), the specific growth rate (µc in h^−1^), the doubling time (DT in h), and the final biomass (FB in CFU/mL).

Table 1 shows that the duration of the Lag phase (adaptation phase of the microorganism to the culture medium) in the spontaneous fermentation (LSF15%) conducted in medium with 15% glucose was high (24 h); in all the inoculated fermentations, the duration of this growth parameter was 0 h.

The LSF15% growth rate (0.095 h^−1^) was identical to that obtained in the medium with 10% glucose (0.097 h^−1^). In the LYF15% and LYF20% tests, the specific growth rates were similar and higher than the others (0.130 and 0.133 h^−1^, respectively). The results suggest that these sugar concentrations stimulated growth. However, the use of 30% glucose inhibited the specific growth rate (0.096 h^−1^). This is in line with reports in the literature that *S. cerevisae* is a Crabtree-negative yeast (repression of respiratory metabolism by glucose) [31]. The doubling times (DT) for LYF15% and LYF20% were shorter (5.32 and 5.21 h), as expected, because DT is inversely proportional to µ_c_.

#### 3.1.2. Solid Media Fermentation

To study solid-medium fermentation, ammonium nitrate (NH_4_NO_3_) was incorporated into the yeast growth medium with the aim of providing better growth conditions for the yeast [21]. Indeed, nitrogen (N) is an essential nutrient for yeast growth and metabolism, and it is fundamental for good fermentative performance [32]. According to Dulf et al. [21], it was possible to increase the antioxidant power and the content of total phenols in fermentations of plum grains in solid medium with the addition of NH_4_NO_3_.

**Figure 4:**
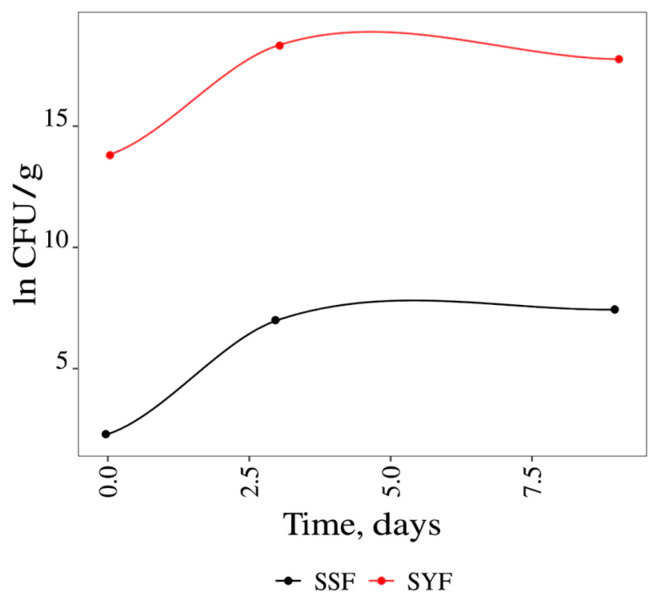
Fermentation results in solid media in ln CFU/g for 9 days. SSF = spontaneous fermentation in solid media; SYF = solid media yeast fermentation.

In Figure 4, the results of microbial growth in ln CFU/g over time (9 days) of solid spontaneous fermentation (SSF) and solid yeast fermentation (SYF) are shown. From the analysis of the results, it can be observed that after 50 h of incubation, the microorganisms entered the stationary phase. The specific growth rate (µ_c_ in h^−1^), doubling time (DT in h), and final biomass (FB in CFU/mL) were also determined (shown in Table S1, Supplementary Materials). The results suggest that in the fermentations conducted in solid media, the duration of the latency phase (Lag) was the same in SSF and SYF. The SYF conducted using *S. cerevisiae* obtained a final biomass of 18.32 ln CFU/g, while in the case of solid spontaneous fermentation (SSF), the maximum growth was 7.43 ln CFU/g. The growth rates and, consequently, doubling times were identical in both cases (Figure 4). In fact, as described in the literature, adequate supplementation of nitrogen sources such as ammonia and amino acids can improve the fermentation process, as it is essential for yeast growth and metabolism, influencing the production of bioactive compounds. In addition, nitrogen affects fermentation kinetics and cell productivity, making it essential for the quality and yield of fermentation processes [33,34]. Ruiz et al. [35] reported that nitrogen is an essential nutrient during wine fermentation because its deficiency causes sullage and tarring in fermentations.

### 3.2. Characterization of V. vinifera L. Leaves and Fermentation Products

#### 3.2.1. Physicochemical Properties

The grapevine leaves were dried, resulting in a loss of 69.90% of water and a color change to dark green. Reduced humidity in relation to grapevine leaves leads to increased quality and stability, thus reducing the rate of microbial deterioration [36]. The analysis of ash, resulting from the complete incineration of plant material found in vine leaves, averaged 3.90%, a value higher than that found by Lima [9] in leaves of the Touriga Franca grape varieties in natura (2.13%). The percentage of fat found in the leaves was 3.30%, close to the values found by Lima [9] in leaves of the Malvasia Fina grape varieties in natura (4.59%) and after bleaching (4.54%). The values obtained in the other samples analyzed by this researcher were higher than 5.00%.

Table 2 shows the results obtained from the physicochemical analyses (ash and fat content) of the leaves of *V. vinifera* “in natura” and the products resulting from the various fermentation processes.

Table 2 shows that the percentage of ash in the solid fermented products, SSF and SYF, was lower than the ash content found in the leaves not subject to fermentation (5.86%). This decrease in the percentage of ash may be related to the use of some substrates during fermentation by the microorganisms that conducted it. However, the content of completely incinerated material of plant origin observed in fermentation in liquid medium (LYF) was higher (37.19%), probably since the speed of growth and the final biomass of the yeasts were higher when growth took place in liquid medium.

The analysis of total lipids showed a high percentage of fats in the vine leaf sample (3.30%) compared to the content found in the fermented leaves (0.48–0.85%) and this difference was significant (*p* < 0.05). However, the fermentations (SSF, SYF, and LYF) showed no significant difference. The decrease in lipid content in the yeast-inoculated trials is probably related to yeast growth; in fact, the lack of long-chain fatty acids and sterols is one of the main causes of difficult fermentations and increased volatile acidity in fermented products.

#### 3.2.2. Chemical Properties

According to the literature, vine leaf extracts contain a promising phenolic content and antioxidant capacity [37]. The extraction yield of *V. vinifera* leaves with 80% ethanol was 24.52%, and the extract was analyzed to determine the total phenols and flavonoid content and the antioxidant activity. The analytical results obtained are presented in Table 3. The results show a total phenol content of 32.32 ± 3.64 mg gallic acid equivalent (GAE)/g of sample. According to the literature, the value of total phenols found in fresh grapevine leaves by Lima [9] was between 139.0 and 170.0 mg GAE/g of extract. The divergence found in this research may have been influenced by the post-harvest processing conditions, namely the drying process to which the leaves were subjected, as well as the time of harvest, climate, and soil, which directly interfere with the composition and quantity of bioactive compounds in the leaves [38].

The flavonoid content observed in this study was 17.41 ± 2.68 mg QE/g, equivalent to that reported by Loizzo et al. [38], which ranged from 2.20–26.2 mg QE/g. This variation can be explained by the fact that environmental factors, genotypes, and post-harvest processing directly influence the content of bioactive compounds present in the vine. This finding corroborates the study by Dhull et al. [16], which demonstrates that solid-state fermentation conducted by *Aspergillus awamori* is a process capable of significantly increasing the total phenolic content and the condensed tannin content.

The result of the analysis of this capacity evaluated by the DPPH method was 37.65 ± 2.18) mg TroloxE/g (51.27%). Our results were identical to those reported by Katalinic et al. [39] and Loizzo et al. [38]. These researchers obtained inhibition values of 34.6–75.4% and 7.19–30.28%, respectively, in different varieties of *V. vinifera*. The result obtained when determining the antioxidant activity using the FRAP method was 79.34 ± 6.74 mg Fe(II)E/g; this value corroborates those found by Loizzo et al. [38], who obtained values of 67.06–100.41 mg Fe(II)E/g.

The results of the total phenolic content of the vine leaves and fermentation products (SSF, SYF, and LYF) are shown in Table 4.

Analyzing the evaluation results of total phenols, fermentation caused the phenolic content of the samples to increase. All the fermentations had a higher phenolic content than the leaves as they were. The spontaneous fermentation showed a phenolic content of 41.62 (±0.57) mg GAE/g, while the fermentations inoculated with *S. cerevisiae* showed a content of 52.58 (±0.35) and 39.23 (±1.85) mg GAE/g in the solid and liquid fermentations, respectively. All fermentations showed significant differences compared to the raw leaf sample. However, the total phenolic contents of SSF and SYF were similar (*p* > 0.05). SYF had the highest phenolic content. These results only prove what has already been mentioned by Dhull et al. [16] and Dulf et al. [21], that fungi and yeasts during fermentation produce hydrolytic enzymes such as beta-glucosidase that catalyze and release aglycones, increasing phenolic compounds, anthocyanins, and antioxidant capacity [16,21]. Studies conducted by Dhull et al. [16] have shown that fermentation can induce the hydrolysis of polymers capable of releasing phenolic compounds from the walls of plant matrices, making them soluble, allowing for an increase in their concentration and the antioxidant potential of the extracts. In addition, the species of microorganism also influences the increase in phenolic content produced during fermentation. *S. cerevisiae* strains produce enzymes such as beta-glucosidases, carboxylesterases and feruloyl esterases, which are effective in releasing insoluble bound phenolics.

#### 3.2.3. Microbiological Analysis Results

To determine the microbiological quality of the leaves subjected to different fermentation processes, the following parameters were assessed: mold and yeast count, lactic acid bacteria count, sulfite-reducing clostridial spore count, *E. coli*/coliform count and coagulase-positive staphylococci count. Table 5 summarizes the results of the mold and yeast count of the *V. vinifera* leaves and fermented products at 1, 3, and 9 days.

Table 5 shows that the mold and yeast counts increased during all the leaf fermentation processes. As expected, the fermentations inoculated with *S. cerevisiae* (SYF and LYF) showed higher mold and yeast counts throughout the fermentation process (1, 3, and 9 days) compared to spontaneous fermentation (SSF). LYF showed marked growth throughout the fermentation process, with colony-forming unit (CFU/g) content varying from 3.85 ± 0.17 log CFU/g to 7.36 ± 0.64 log CFU/g.

Sulfite-reducing clostridial spores were absent in all the samples analyzed. The *E. coli*/Total coliform and coagulase-positive Staphylococci count was 1 log CFU/mL in all samples.

### 3.3. Yogurt Analysis

#### 3.3.1. Physicochemical Analysis Results of the Yogurt Samples

The physicochemical characteristics (ash, fat, protein, pH, and titratable acidity) of the four yogurt samples were determined: the control with the addition of potassium sorbate (Control yogurt, Cy) and the other three with the incorporation of vine leaf fermentation extract (SSFy, SYFy, and LYFy), at storage times of 1, 7, and 12 days at 4 °C. Table 5 shows the results of the physicochemical composition of the four samples. For each variable, the comparison of means showed that there were no significant differences.

The results in Table 6 show that the ash, fat, protein, pH, and titratable acidity content of the four yogurt samples (Cy, SSFy, SYFy, and LYFy) did not differ significantly (*p* > 0.05) from each other. Overall, the carbohydrate content was about 18.6% (calculated value according to AOAC Official Method 986.25.) for each yogurt sample. The ash content was similar to the values observed by Santos et al. [29], who observed 0.71% in terms of Cy, and Afiyah et al. [40], who noted 0.82%. In our study, ash values ranging from 0.77 - 0.69% were observed in terms of Cy, while values ranging from 0.58–0.94% were observed in yogurts with incorporated vine leaf extracts (SSFy, SYFy, and LYFy). Santos et al. [29] observed values of 0.70% for this parameter in yogurt containing red propolis, while Afiyah et al. [40] obtained results ranging from 0.75–0.79% in yogurt containing mango juice (*Mangifera indica* L.). The lipid content ranged from 2.00–2.27%, which is lower than the values found by Santos et al. [29], Afiyah et al. [40] and Haq et al. [41] in yogurt supplemented with lentil flour. The lower fat content observed in this work may be a consequence of the ingredients used to prepare it, such as low-fat milk powder and low-fat pasteurized milk. According to European legislation, our yogurt would be classified as semi-skimmed [42]. The protein content of the yogurts studied ranged from 2.15–2.63%, which was slightly lower than those reported by Santos et al. [29], Haq et al. [41], and Fagnani and Boniatti [42] enriched with grape seed flour. None of the yogurt samples evaluated showed significant differences (*p* > 0.05) in terms of pH values. The pH values found ranged from 4.16–4.34. It should be noted that the control sample had the highest pH values at all the times analyzed. All the samples showed a decrease in pH values over time (12 days), which was to be expected since homofermentative microorganisms, such as *Lactobacillus acidophilus*, continue to ferment lactose and reduce the pH by forming lactic acid (LA) [43]. The titratable acidity of the yogurts ranged from 0.90–1.08 g of LA/100 g. All the samples showed a slight variation in titratable acidity over the storage period. However, it is believed that over the course of the yogurt’s shelf life, all the samples would show an increase in acidity, which would be advantageous for the yogurt since acidity helps to control the undesirable growth of pathogenic and spoiling bacteria [44]. It was found that replacing potassium sorbate (Cy) with vine leaf extract (SSFy, SYFy, and LYFy) did not significantly alter the physicochemical composition of yogurt samples.

#### 3.3.2. Chemical Analysis Results of the Yogurt Samples

The results of the analyses of total phenols and antioxidant activity (DPPH and FRAP) of the yogurts at 1 and 12 days of storage are summarized in Table 7.

The total phenol content and antioxidant activity observed in the yogurts were higher in the samples with added *V. vinifera* leaf extract (SSFy, SYFy, and LYFy) compared to the control (Cy). It was found that in all the samples, the total phenol content was higher at 12 days of storage. The significant increase in total phenolics corroborates the achievement of the objectives outlined in this study. The observed improvement can be directly attributed to the incorporation of fermented extracts into yogurt formulations, highlighting their direct influence on the antioxidant properties of dairy products.

The yogurt sample with the highest phenol content was SYFy (2.16 ± 0.28 mg GAE/g of yogurt). In addition, it was the only sample that showed a significant difference (*p* < 0.05) between 1 and 12 days. The results of the analysis of antioxidant activity using the DPPH method showed that none of the samples presented any statistical difference. The LYFy sample was the only one with an increase in antioxidant content using the DPPH method, from 1.19 ± 0.34–1.38 ± 0.06 mg TroloxE/g of yogurt. The FRAP results showed that only the control (Cy) had a reduced antioxidant content (from 4.22 ± 0.16–4.00 ± 0.36 mg Fe(II)E/g of yogurt), while the other yogurt samples (SSFy, SYFy, and LYFy) had increased antioxidant content, particularly the SYFy sample, which was the only one to show a significant difference (from 5.28 ± 0.25–5.77 ± 0.21 mg Fe(II)E/g of yogurt). In yogurt containing added vine leaf extracts, especially the fermented ones, the phenolic content and the antioxidant activity were higher than in the control. This suggests that these compounds probably originated from leaf extracts, which may increase the bioactive properties of the final product. This shows that the addition of the leaf extract makes it possible to obtain foods with higher nutritional quality and health benefits due to their antioxidant and antimicrobial properties [11].

#### 3.3.3. Microbiological Analysis Results of the Yogurt Samples

Over its shelf life, yogurt is susceptible to microbiological changes. The conditions in which the product is stored interfere with its microbial stability, particularly temperature, pH, oxygen, and the energy source (carbohydrates, proteins, and fats). In addition, the growth of deteriorating microorganisms, such as coliforms, molds and yeasts, can lead to changes in the appearance, aroma, and taste of the yogurt, as well as indicating that sanitary practices were poor during production [35]. Antimicrobial additives used in food products are necessary to inhibit these types of microorganisms [45]. The effect of the extracts added to yogurt corroborates the studies carried out by De Andrade et al. [46] and Katalinic et al. [39], which indicate that the bioactive compounds present in vine leaves have antioxidant and antimicrobial potential since these compounds induce metabolic effects that are beneficial to health and offer possibilities for their application in the prevention of oxidative and/or microbial deterioration of food products. The results obtained for the lactic acid bacteria in yogurts with strawberry pulp on days 1, 7, and 12 of storage are shown in Table 8.

**Figure 5:**
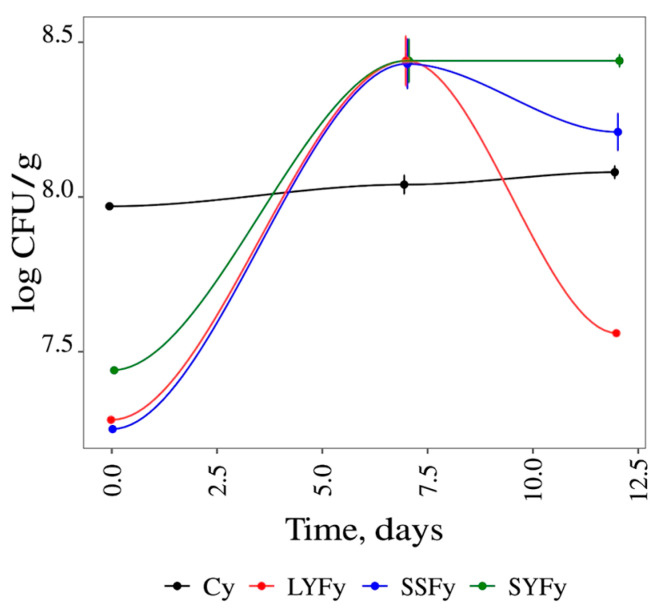
Lactic bacteria count of yogurt samples during 12 days of storage.

The results of the microbiological analysis showed an absence of *E. coli*/total coliforms, in accordance with the European parameters for the microbiological quality of yogurt. According to the microbiological criteria of the European Union, all samples showed count values of <10 CFU/mL for molds and yeasts, sulphite-reducing clostridia and coagulase-positive *Staphylococcus* [47]. The absence of these microorganisms is an indication of good practices during the production of this product. The lactic acid bacteria count was stable in all the yogurt samples. Figure 5 illustrates the behavior of lactic acid bacteria during the 12 days of evaluation.

The lactic acid bacteria count in the control varied from 7.97 ± 0.01–8.08 ± 0.05 log CFU/g over the 12 days of storage (not significant mean differences). The SSFy, SYFy, and LYFy samples had a maximum count of 8.43 ± 0.19, 8.44 ± 0.15 and 8.44 ± 0.18, respectively, at 7 days. At 12 days of storage, the amount of these bacteria decreased slightly in the SSFy sample (8.21 ± 0.13 log CFU/g) and significantly decreased in the LYFy sample (7.56 ± 0.01 log CFU/g) compared to the results at 7 days. Meanwhile, SYFy maintained a count of 8.44 log CFU/g from 7 to 12 days of storage. In this context, it is considered that introducing or replacing an additive in yogurt helps preserve and stabilize the product, as well as enabling the growth of lactic acid bacteria.

## 4. Conclusions

In this study, we characterized the fermentative process of *V. vinifera* L. leaves (grapevine). We then evaluated the effects on bioactivity and shelf life by mixing the fermented leaves with yogurt, exploring their potential role as preserving agents.

The replacement of potassium sorbate preservative in yogurts by fermented grapevine leaf extract increased the phenolic content, enhancing its antioxidant activity. Microbiological evaluation indicated that grapevine leaf fermentation extract possesses antimicrobial potential without interfering with the growth of lactic acid bacteria. Among the fermentations, the one conducted in a solid medium demonstrated the best performance and enhanced biological properties. Additionally, yogurts incorporating the extract showed no significant differences in physicochemical properties, pH, or acidity compared to the control. Therefore, we provide evidence that may allow taking advantage of an agricultural by-product to increase the quality and nutritional value of a widely consumed food product.

As the demand for natural additives continues to increase, the products developed could be considered an alternative source to introduce into the food additives market. However, future research should assess the sensory aspect and toxicity of this natural additive, which is currently a highly abundant and wasted residue. Additionally, it will be relevant to evaluate the antimicrobial activity of fermented grapevine leaves against specific spoilage microorganisms (challenge test).

## Figures and Tables

**Table 1:** Growth parameters of *S. cerevisiae* across the various study conditions in liquid media.

Liquid Medium Fermentation				
Sample	Lag Phase (h)	µ_c_ (h^−1^)	DT (h)	FB (CFU/mL)
LSF15%	24	0.095	7.27	4.80 × 10^7^ ± 0.00
LYF10%	0	0.097	7.18	3.40 × 10^7^ ± 0.04
LYF15%	0	0.130	5.32	3.80 × 10^8^ ± 0.04
LYF20%	0	0.133	5.21	3.40 × 10^7^ ± 0.05
LYF30%	0	0.096	7.23	2.70 × 10^7^ ± 0.32

LSF15% = Liquid spontaneous fermentation with 15% glucose; LYF10%: = liquid yeast fermentation with 10% glucose; LYF15% = liquid yeast fermentation with 15% glucose; LYF20% = liquid yeast fermentation with 20% glucose; LYF30% = liquid yeast fermentation with 30% glucose; Lag phase = adaptation time (h); µ_c_ = specific rate of growth (h^−1^); DT = duplication time (h); FB = final biomass (CFU/mL).

**Table 2:** Results of the physicochemical analysis (ash and fat content) of the leaves of *V. vinifera* “in natura” and after the various fermentation processes.

Assay	Ash (%)	Fat (%)
Leaf	5.86 ± 0.69 ^a^	3.30 ± 0.03 ^a^
SSF	1.21 ± 0.83 ^b^	0.85 ± 0.15 ^b^
SYF	2.22 ± 0.33 ^b^	0.80 ± 0.26 ^b^
LYF	37.19 ±1.01 ^c^	0.48 ± 0.28 ^b^

Means followed by qual letters are not statistically different among themselves by Tukey test at 5% probability. SSF = solid spontaneous fermentation; SYF = solid yeast fermentation; LYF = liquid yeast fermentation. In each variable, different letters indicate significant mean differences (a–c).

**Table 3:** Total phenol and flavonoid contents and antioxidant activity (DPPH and FRAP) of the grapevine leaves.

	Total Phenols mg GAE/g	Total Flavonoids mg QE/g	DPPH mg TroloxE/g	FRAP mg Fe(II)E/g
**Leaves of *V. vinifera***	32.32 ± 3.64	17.41 ± 2.68	37.65 ± 2.18	79.34 ± 6.74

Results expressed in mean of analysis ± standard deviation. GAE: gallic acid equivalent; QE: quercetin equivalent; DPPH: 2,2-Diphenyl-1-picrylhydrazyl; FRAP: ferric-reducing antioxidant power.

**Table 4:** Total phenol content of the leaves of *V. vinifera* and of the leaf fermentations.

Assay	Total Phenol Content mg GAE/g
**Leaf**	32.32 ± 3.64 ^a^
**SSF**	41.62 ± 0.57 ^b^
**SYF**	52.58 ± 0.35 ^c^
**LYF**	39.23 ± 1.85 ^b^

Results expressed in mean ± standard deviation. Means followed by the same letters are not statistically different using the Tukey test at 5% probability. GAE: gallic acid equivalent; SSF: spontaneous fermentation in solid media; SYF: yeast fermentation in solid media; LYF: yeast fermentation in liquid media. Different letters indicate significant mean differences (a–c).

**Table 5:** Total enumeration of molds and yeasts in grapevine leaves and throughout the fermentation process.

Days	Yogurt	Mold and Yeast (log CFU/mL)
1	Leaf	1.00 ± 0.07 ^a^
	SSF	1.00 ± 0.01 ^a^
	SYF	2.16 ± 0.08 ^b^
	LYF	3.85 ± 0.17 ^c^
3	SSF	2.26 ± 0.07 ^b^
	SYF	4.11 ± 0.21 ^c^
	LYF	4.87 ± 0.55 ^c^
9	SSF	1.18 ± 0.20 ^a^
	SYF	6.36 ± 0.38 ^d^
	LYF	7.36 ± 0.64 ^d^

Means followed by the same letters do not differ statistically from each other according to the Tukey test at a 5% probability level. SSF = solid spontaneous fermentation; SYF = solid yeast fermentation; LYF = liquid yeast fermentation. Different letters indicate significant mean differences (a–d).

**Table 6:** Physicochemical properties of the yogurts at different timepoints (1, 7, and 12 days).

Day	Yogurt	Ash (%)	Fat (%)	Protein (%)	pH	TA g of LA/100 g
**1**	Cy	0.75 ± 0.04	2.14 ± 0.04	2.63 ± 0.05	4.34 ± 0.04	1.08 ± 0.02
	SSFy	0.69 ± 0.15	2.07 ± 0.17	2.51 ± 0.08	4.18 ± 0.10	0.98 ± 0.05
	SYFy	0.94 ± 0.07	2.00 ± 0.12	2.15 ± 0.04	4.24 ± 0.02	0.93 ± 0.08
	LYFy	0.69 ± 0.01	2.12± 0.07	2.48 ± 0.04	4.26 ± 0.01	1.05 ± 0.07
**7**	Cy	0.77 ± 0.02	2.09 ± 0.05	2.48 ± 0.11	4.34 ± 0.05	0.95 ± 0.07
	SSFy	0.71 ± 0.05	2.12 ± 0.13	2.48 ± 0.13	4.20 ± 0.03	0.95 ± 0.07
	SYFy	0.58 ± 0.11	2.20 ± 0.06	2.51± 0.07	4.25 ± 0.03	0.98 ± 0.05
	LYFy	0.71 ± 0.02	2.24 ± 0.01	2.43 ± 0.10	4.19 ± 0.07	0.93 ± 0.06
**12**	Cy	0.69 ± 0.10	2.15 ± 0.03	2.53 ± 0.02	4.28 ± 0.07	0.94 ± 0.00
	SSFy	0.72 ± 0.01	2.27 ± 0.02	2.84 ± 0.06	4.16 ± 0.05	1.00 ± 0.04
	SYFy	0.72 ± 0.12	2.16 ± 0.10	2.93 ± 0.10	4.20 ± 0.00	0.90 ± 0.04
	LYFy	0.75 ± 0.03	2.25 ± 0.14	2.80 ± 0.07	4.19 ± 0.00	0.97 ± 0.04

TA = titratable acidity; LA = lactic acid; Cy = control yogurt; SSFy = yogurt with an extract from solid spontaneous fermentation; SYFy = yogurt with an extract from solid yeast fermentation; LYFy = yogurt with an extract from liquid yeast fermentation.

**Table 7:** Total phenolic content and antioxidant activity (DPPH and FRAP) of yogurts at 1 and 12 days of storage.

Days	Yogurt	Total Phenols mg GAE/g	DPPH mg TroloxE/g	FRAP mg Fe(II)E/g
1	Cy	1.23 ± 0.05 ^a^	1.19 ± 0.34 ^a^	4.22 ± 0.16 ^a^
	SSFy	1.60 ± 0.13 ^b^	1.23 ± 0.06 ^a^	5.06 ± 0.19 ^b^
	SYFy	1.87 ± 0.01 ^b^	1.51 ± 0.07 ^a^	5.28 ± 0.25 ^b^
	LYFy	1.34 ± 0.13 ^ab^	1.19 ± 0.13 ^a^	3.80 ± 0.27 ^a^
12	Cy	1.44 ± 0.07 ^ab^	1.01 ± 0.06 ^a^	4.00 ± 0.36 ^a^
	SSFy	1.94 ± 0.14 ^b^	1.33 ± 0.06 ^a^	5.30 ± 0.12 ^b^
	SYFy	2.16 ± 0.28 ^c^	1.34 ± 0.08 ^a^	5.77 ± 0.21 ^c^
	LYFy	1.43 ± 0.11 ^ab^	1.38 ± 0.06 ^a^	4.32 ± 0.33 ^a^

Results expressed in mean of the analyses ± standard deviation. Means followed by same letters are not statistically different using the Tukey test at 5% probability. GAE = gallic acid equivalent; DPPH: 2,2-Diphenyl-1-picrylhydrazyl; FRAP = ferric-reducing antioxidant power; Cy = control yogurt; SSFy = yogurt with extract from solid spontaneous fermentation; SYFy = yogurt with extract from solid yeast fermentation; LYFy = yogurt with extract from liquid yeast fermentation. In each variable, different letters indicate significant mean differences (a–c).

**Table 8:** Lactic bacteria count of samples at different storage times (1, 7, and 12 days).

Days	Yogurt	Lactic Bacteria log CFU/mL
1	Cy	7.97 ± 0.01 ^b^
	SSFy	7.25 ± 0.01 ^c^
	SYFy	7.44 ± 0.02 ^c^
	LYFy	7.28 ± 0.01 ^c^
7	Cy	8.04 ± 0.07 ^b^
	SSFy	8.43 ± 0.19 ^a^
	SYFy	8.44 ± 0.15 ^a^
	LYFy	8.44 ± 0.18 ^a^
12	Cy	8.08 ± 0.05 ^b^
	SSFy	8.21 ± 0.13 ^ab^
	SYFy	8.44 ± 0.05 ^a^
	LYFy	7.56 ± 0.01 ^c^

Cy = control yogurt; SSFy = yogurt with extract from solid spontaneous fermentation; SYFy = yogurt with extract from solid yeast fermentation; LYFy = yogurt with extract from liquid yeast fermentation. Different letters indicate significant mean differences (a–c).

## Data Availability

The original contributions presented in the study are included in the article/Supplementary Material, further inquiries can be directed to the corresponding author.

## Supplementary Materials

The following supporting information can be downloaded at: https://www.mdpi.com/article/10.3390/foods13132053/s1. Figure S1. Evolution of the weights of the flasks in the fermentations in liquid media with 15% and 20% of glucose. LSF15% = Liquid spontaneous fermentation with 15% of glucose; LSF20%= Liquid spontaneous fermentation with 20% of glucose; LYF15% = Liquid yeast fermentation with 15% of glucose; LYF20% = Liquid yeast fermentation with 20% of glucose; Figure S2. Soluble solids of the fermentations in liquid media with 15% and 20% of glucose. LSF15% = Liquid spontaneous fermentation with 15% of glucose; LSF20% = Liquid spontaneous fermentation with 20% of glucose; LYF15% = Liquid yeast fermentation with 15% of glucose; LYF20%: Yeast fermentation with 20% of glucose; Figure S3. Soluble solids of the fermentations in liquid media with 15% of glucose. LSF15% = Liquid spontaneous fermentation with 15% of glucose; LYF15% = Liquid yeast fermentation with 15% of glucose; Table S1. Growth parameters of S. cerevisiae in solid media.

## References

[1] Mathias T.R.S., Carvalho Junior I.C., Carvalho C.W.P., Sérvulo E.F.C. (2011). Rheological characterization of coffee-flavored yogurt with different types of thickener. Alim. Nutr..

[2] Lopes R.P., Mota M.J., Pinto C.A., Souza S., Silva J.A.L., Gomes A.M., Delgadillo I., Saraiva J.A. (2019). Physicochemical and microbial changes in yogurts produced under different pressure and temperature conditions. LWT.

[3] Santos J.V.R., Miranda E.S.M., Oliveira A.T.C., Damaceno M.N., Silva M.S., Cavalcante A.B.D. (2020). Cinética da fermentação de leite adicionado de farinha de banana verde na produção de iogurte. Res. Soc. Dev..

[4] Kefi B.B., Baccouri S., Torkhani R., Koumba S., Martin P., M’hamdi N. (2022). Application of response surface methodology to optimize solid-phase extraction of benzoic acid and sorbic acid from food drinks. Foods.

[5] Zhang W., Yang F., Xu J., Wang L., Zhou K. (2021). Determination of nine preservatives in food samples by solid phase extraction coupled with capillary electrophoresis. Int. J. Electrochem. Sci..

[6] Dey S., Nagababu B.H. (2022). Applications of food color and bio-preservatives in the food and its effect on the human health. Food Chem..

[7] European Commission (2011). Commission Regulation (EU) No 1129/2011 of 11 November 2011 Amending Annexes II and III to Regulation (EC) No 1333/2008 of the European Parliament and of the Council by Establishing a Union List of Food Additives. https://eur-lex.europa.eu/legal-content/PT/TXT/PDF/?uri=CELEX:32011R1129&from=es.

[8] Sat I.G., Sengul M., Keles F. (2002). Use of grape leaves in canned food. Pak. J. Nutr..

[9] Lima A.F. (2015). Caracterização da Bioatividade de Folhas de Diferentes Castas de Videira Quando Sujeitas a Processamento Alimentar. Master’s Thesis.

[10] Nzekoue F.K., Nguefang M.L.K., Alessandroni L., Mustafa A.M., Vittori S., Caprioli G. (2022). Grapevine leaves (*Vitis vinifera*): Chemical characterization of bioactive compounds and antioxidant activity during leave development. Food Biosci..

[11] Sahu A., Singh D., Shukla R. (2023). Bioactive compounds and reported pharmacological activities of *Vitis vinifera* L.—An overview. World J. Pharm. Res..

[12] Caleja C.S.G. (2018). Incorporação de Ingredientes Naturais em Diferentes Matrizes Alimentares como Potenciadores de Conservação e Promotores de Saúde. Ph.D. Thesis.

[13] Liu N., Song M., Wang N., Wang Y., Wang R., An X., Qi J. (2020). The effects of solid-state fermentation on the content, composition and in vitro antioxidant activity of flavonoids from dandelion. PLoS ONE.

[14] Lee B.H. (1996). Fundamentos de Biotecnología de los Alimentos.

[15] Rhodes A., Fletcher D.L. (1975). Principles of Industrial Microbiology.

[16] Dhull S.B., Punia S., Kidwai M.K., Kaur M., Chawla P., Purewal S.S., Sangwan M., Palthania S. (2020). Solid-state fermentation of lentil (*Lens culinaris* L.) with *Aspergillus awamori*: Effect on phenolic compounds, mineral content, and their bioavailability. Legume Sci..

[17] Xu L., Guo S., Zhang S. (2018). Effects of solid-state fermentation with three higher fungi on the total phenol contents and antioxidante properties of diverse cereal grains. FEMS Microbiol. Lett..

[18] García L.R.P., Bianchi V.L.D. (2015). Efeito da fermentação fúngica no teor de compostos fenólicos em casca de café robusta. Semin. Ciências Agrárias.

[19] Kosar M., Küpeli E., Malyer H., Uylaser V., Türkben C., Baser K.H.C. (2007). Effect of brining on biological activity of leaves of *Vitis vinífera L.* (Cv. Sultani Cekirdeksiz) from Turkey. J. Agric. Food Chem..

[20] Ferreira L.M.M., Ferreira A.M., Benevides C.M.J., Melo D., Costa A.S.G., Faia A.M., Oliveira M.B.P.P. (2019). Effect of controlled microbial fermentation on nutritional and functional characteristics of cowpea bean flours. Foods.

[21] Dulf F.V., Vodnar D.C., Socaciu C. (2016). Effects of solid-state fermentation with two filamentous fungi on the total phenolic contents, flavonoids, antioxidant activities and lipid fractions of plum fruit (*Prunus domestica* L.) by-products. Food Chem..

[22] Paula V.M.B. (2012). Caraterização Química e Biológica do Própolis da “Serra de Bornes” por TLC. Master’s Thesis.

[23] Celik O.F., Temiz H. (2022). Lactobacilli isolates as potential aroma producer starter cultures: Effects on the chemical, physical, microbial, and sensory properties of yogurt. Food Biosci..

[24] Muncan J., Tei K., Tsenkova R. (2020). Real-time monitoring of yogurt fermentation process by aquaphotomics near-infrared spectroscopy. Sensors.

[25] Water J.V., Naiyanetr P. (2003). Yoghurt and immunity: The health benefits of fermented milk products that contain lactic acid bacteria. Handbook of Fermented Functional Foods.

[26] AOAC (2012). Association of Official Analytical Chemistry: Official Methods of Analysis.

[27] Singleton V.L., Orthofer R., Lamuela-Raventos R.M. (1999). Analysis of total phenols and other oxidation substrates and antioxidants by means of Folin–Ciocalteu reagent. Methods Enzymol..

[28] Savi P.R.S., Santos L., Gonçalves A.M., Biesek S., Lima C.P. (2017). Análise de flavonoides totais presentes em algumas frutas e hortaliças convencionais e orgânicas mais consumidas na região sul do Brasil. Demetra Aliment. Nutr. Saúde.

[29] Santos M.S., Estevinho L.M., Carvalho C.A.L., Morais J.S., Conceição A.L.S., Paula V.B., Guedes K.M., Almeida R.C.C. (2019). Probiotic yogurt with brazilian red propolis: Physicochemical and bioactive properties, stability, and shelf life. J. Food Sci..

[30] Liofilchem (2015). Rose Bengal CAF Agar. Selective Medium for Detection of Yeasts and Moulds from Food and Environmental Materials. http://www.liofilchem.net/login/pd/ifu/10034_IFU.pdf.

[31] Dai Z., Huang M., Chen Y., Siewers V., Nielsen J. (2018). Global rewiring of cellular metabolism renders *Saccharomyces cerevisiae* Crabtree negative. Nat. Commun..

[32] Mendes-Ferreira A., Sampaio-Marques B., Barbosa C., Rodrigues F., Costa V., Mendes-Faia A., Ludovico P., Leao C. (2010). Accumulation of non-superoxide anion reactive oxygen species mediates nitrogen-limited alcoholic fermentation by *Saccharomyces cerevisiae*. Appl. Environ. Microbiol.

[33] Vidal E.E. (2012). Influência da Fonte de Nitrogênio no Perfil Fermentativo, Transcriptômico, e na Produção de Álcoois Superiores em *Saccharomyces cerevisiae*. Master’s Thesis.

[34] Pereira A.F. (2007). Suplementação de Nitrogênio Sobre a Fermentação Alcoólica para Produção de Cachaça, Cerveja e Vinho. Master’s Thesis.

[35] Ruiz J., Celis M., Toro M., Mendes-Ferreira A., Rauhut D., Santos A., Belda I. (2020). Phenotypic and transcriptional analysis of *Saccharomyces cerevisiae* during wine fermentation in response to nitrogen nutrition and co-inoculation with *Torulaspora delbrueckii*. Int. Food Res..

[36] Câmara G.B., Oliveira T.K.B., Macedo C.D.S., Leite D.D.D.F., Soares T.D.C., Lima A.R.N., Vasconcelos S.H., Soares T.C., Barbosa M.L., Trigueiro L.S.D.L. (2019). Physico-chemical, toxicological and nutritional characterization of dry and in *natura Moringa oleifera Lam* leaves. Res. Soc. Dev..

[37] Fernandes F., Ramalhosa E., Pires P., Verdial J., Valentão P., Andrade P., Bento A., Pereira J.A. (2013). *Vitis vinífera* leaves towards bioactivity. Ind. Crops Prod..

[38] Loizzo M.R., Sicari V., Pellicanò T., Xiao J., Poiana M., Tundis R. (2019). Comparative analysis of chemical composition, antioxidant and antiproliferative activities of Italian *Vitis vinifera* by-products for a sustainable agro-industry. Food Chem. Toxicol..

[39] Katalinic V., Mozina S.S., Generalic I., Skroza D., Ljubenkov I., Klancnik A. (2013). Phenolic profile, antioxidant capacity, and antimicrobial activity of leaf extracts from six *Vitis vinífera L*. varieties. Int. J. Food Prop..

[40] Afiyah D.N., Sarbini R.N., Huda M.S. (2022). Analysis of the yogurt nutrient content and antioxidant activity by adding Podang Urang Mango juice (*Mangifera indica* L.). J. Ternak.

[41] Haq F.U., Sameen A., Zaman Q.U., Mushtaq B.S., Hussain M.B., Javed A., Plygun S., Korneeva O., Shariati M.A. (2019). Development and evalutation of yogurt supplemented with lentil flour. JMBFS..

[42] Fagnani R., Boniatti P.M.S. (2020). Formulação de iogurte concentrado enriquecido com farinha de semente de uva: Atividade antioxidante e cinética de fermentação. Ensaios.

[43] Lopes R.P. (2013). Effects of high hydrostatic pressure on yogurt production. Master’s Thesis.

[44] Duarte M.C.K.H., Cortez N.M.S., Cortez M.A.S., Franco R.M., Macedo N. (2016). Ação antagonista de *Lactobacillus acidophilus* frente a estirpes patogênicas inoculadas em leite fermentado. J. Bioenergy Food Sci..

[45] Anari H.N.B., Majdinasab M., Shaghaghian S., Khalesi M. (2022). Development of a natamycin-based non-migratory antimicrobial active packaging for extending shelf-life of yogurt drink (Doogh). Food Chem..

[46] De Andrade R.B., Machado B.A.S., Barreto G.d.A., Nascimento R.Q., Corrêa L.C., Leal I.L., Tavares P.P.L.G., Ferreira E.d.S., Umsza-Guez M.A. (2021). Syrah Grape Skin Residues Has Potential as Source of Antioxidant and Anti-Microbial Bioactive Compounds. Biology.

[47] European Commission (2005). Commission Regulation (EC) No. 2073/2005 of 15 November 2005 on microbiological criteria for foodstuffs. Off. J. Eur. Union.

