# Supplementary material for: Fermented Grapevine Leaves: Potential Preserving Agent in Yogurt
Source: Foods. 2024 Jun 27;13(13):2053. doi: 10.3390/foods13132053 (PMC11241237; doi:10.3390/foods13132053)
Supplement: Supplementary file 1 [file foods-13-02053-s001.zip › foods-3000610-supplementary.pdf]

Supplementary material:

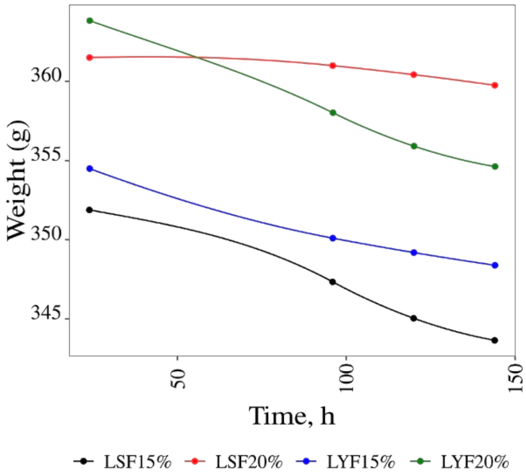

Figure S1. Evolution of the weights of the flasks in the fermentations in liquid media with 15% and 20% of glucose. LSF15%= Liquid spontaneous fermentation with 15% of glucose; LSF20%= Liquid spontaneous fermentation with 20% of glucose; LYF15%= Liquid yeast fermentation with 15% of glucose; LYF20%= Liquid yeast fermentation with 20% of glucose.

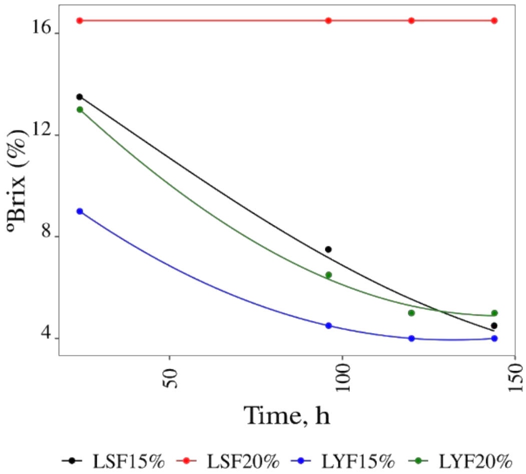

Figure S2. Soluble solids of the fermentations in liquid media with 15% and 20% of glucose. LSF15%= Liquid spontaneous fermentation with 15% of glucose; LSF20%= Liquid spontaneous fermentation with 20% of glucose; LYF15%= Liquid yeast fermentation with 15% of glucose; LYF20%: Yeast fermentation with 20% of glucose.

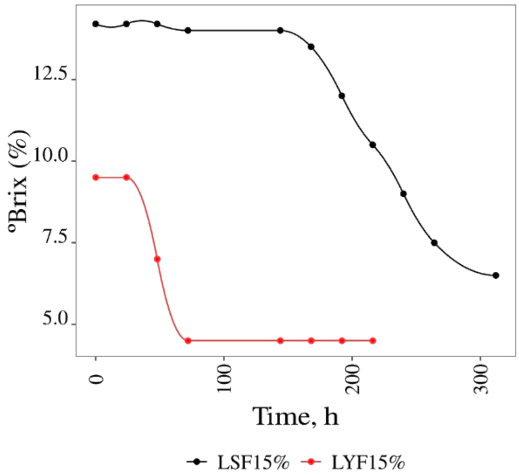

Figure S3. Soluble solids of the fermentations in liquid media with 15% of glucose. LSF15%= Liquid spontaneous fermentation with 15% of glucose; LYF15%= Liquid yeast fermentation with 15% of glucose.

Table S1. Growth parameters of *S. cerevisiae* in solid media.

**Solid medium fermentation**				
Sample	Lag phase (h)	μ_c (h^{-1})	DT (h)	FB (CFU/g)
SSF	0	0.0653	10.61	$1.70 \times 10^3 \pm 0.00$
SYF	0	0.0628	11.04	$8.10 \times 10^7 \pm 0.08$

SSF= Solid spontaneous fermentation; SYF= Solid yeast fermentation; Lag phase= Adaptation time (hours); μ_c= Specific growth rate ($hours^{-1}$); DT= Duplication time (hours); FB= Final biomass (CFU/g).
